# Translating multiscale research in rare disease

**DOI:** 10.1242/dmm.052009

**Published:** 2024-07-10

**Authors:** Kirsty M. Hooper, Monica J. Justice, Monkol Lek, Karen J. Liu, Katherine A. Rauen

**Affiliations:** ^1^ The Company of Biologists, Bidder Building, Station Road, Histon, Cambridge CB24 9LF, UK; ^2^ Program in Genetics and Genome Biology, The Hospital for Sick Children, Toronto, Ontario, M5G 0A4, Canada; ^3^ Department of Genetics, Yale University School of Medicine, New Haven, CT 06510, USA; ^4^ Department of Craniofacial Development and Stem Cell Biology, King's College London Dental Institute, Guy's Hospital, London SE1 9RT, UK; ^5^ Department of Pediatrics, Division of Genomic Medicine, University of California Davis, Sacramento, CA 95817, USA; ^6^ University of California Davis MIND Institute, Sacramento, CA 95817, USA

## Abstract

**Summary:** This Editorial introduces DMM's new Special Issue on ‘Translating Multiscale Research in Rare Disease’. The Guest Editors reflect on how articles in the issue advance the rare disease research field.


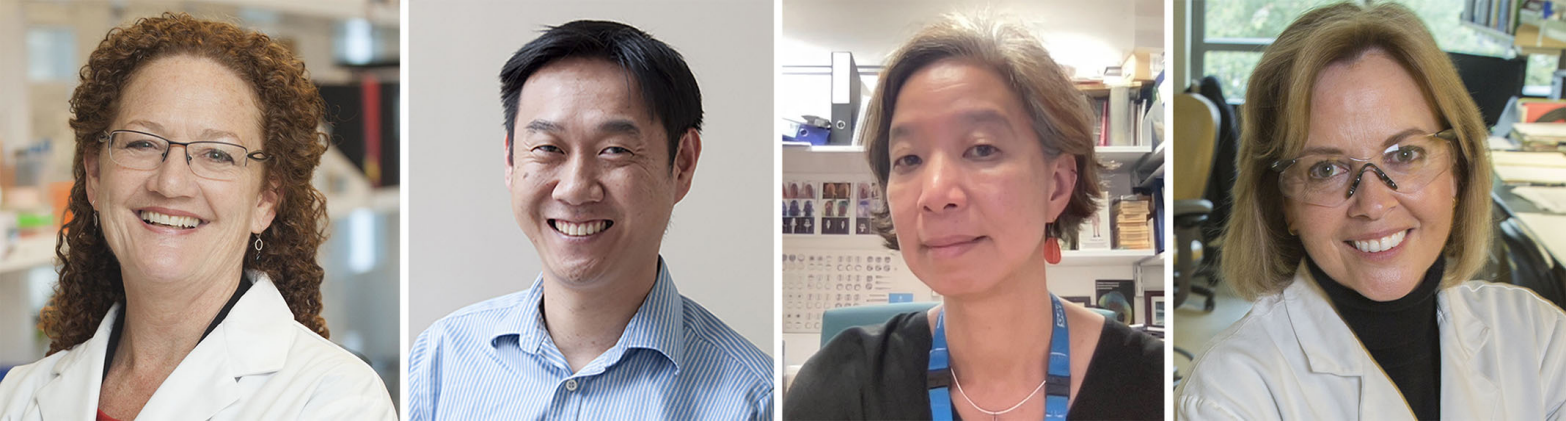


**The Special Issue Editors (left to right): Monica Justice, Monkol Lek, Karen Liu and Kate Rauen**

Rare diseases affect biological function across scales – from individual genes and their products, to tissues, organs and the whole organism. Individually, a rare disease might affect only a few people, making them difficult to recognise, diagnose or treat, but collectively, they affect approximately 400 million people globally (Nguengang Wakap et al., 2020). This underscores the need for pre-clinical research that interrogates the mechanisms of these disorders to foster meaningful clinical progress in their diagnosis and treatment. Our goal in shaping this Special Issue of Disease Models & Mechanisms (DMM) was to compile original Research, Resources & Methods and Review-type articles that focus on the genomics, phenomics, networks, mechanisms and pathways of rare diseases (Hooper and Hmeljak, 2023). In this Editorial, we discuss how the articles in this Special Issue are contributing to the progress of the field.

## The impact of the ‘genetics boom’ on investigating rare disease

The field of genetics has taken astronomical leaps in recent decades. This has accelerated rare disease research and clinical management, as understanding the genetic aetiology of a rare disease can open up avenues for characterising its mechanisms and diagnosing and treating patients. The ability to engineer precise mutations in mouse models, for instance, is changing our view of disease models. In the past, mouse geneticists were restricted to analysing random mutations or knockout loss-of-function mutations generated in embryonic stem cells. Now, with CRISPR/Cas9 genome editing, precise variants found in human patients of many diseases can be recapitulated in a model organism. Marshall et al. (2024) engineered a mouse model of a precise missense variant in *EEF1A2* that is found in a rare neurodevelopmental disorder and causes severe seizures and developmental delay in affected children, and compared the phenotype in these mice with *Eef1a2* knockout mice. Surprisingly, the mice with the precise missense mutation presented with movement difficulties and seizures much earlier than the knockout mice, suggesting some toxic effects of this mutation. Furthermore, the missense mutation affected muscle more than neurons. These findings enable therapies to be developed based on the phenotype of individual variants, a field called precision medicine.

X-linked conditions, which are the disorders associated with gene variants in the X chromosome, can also be precisely modelled in mice. Snyder–Robinson Syndrome (SRS) is a rare X-linked disorder characterized by a number of symptoms that include intellectual disability, muscular and skeletal abnormalities, and seizures in males. SRS is caused by mutation of the enzymes that generate polyamine metabolites, which must be tightly controlled to properly regulate many critical cellular functions. In a study by Akinyele et al. (2024), the authors produced a mouse model by engineering a precise variant found in patients with SRS in the enzyme spermine synthase. The mouse model recapitulated the symptoms found in humans, making it useful for understanding disease progression of spermine-synthase-based SRS. This can inform the clinical management of patients with SRS and provides a first step towards the development of effective therapies.

The neurological disorder Rett syndrome (RTT) is an X-linked condition caused by variants in the epigenetic factor methyl-CpG-binding protein 2 (MECP2), which transcriptionally regulates many genes, among them brain-derived neurotrophic factor (BDNF). Medeiros et al. (2024) tested the effects of a small-molecule ligand for the BDNF receptor in female *Mecp2*/+ mutant mice. Interestingly, this ligand restored neuron morphology and behaviours in the mice, suggesting that this small molecule could be a treatment for RTT, as well as for other BDNF-related neurological disorders.

Duchenne muscular dystrophy (DMD) is a rare X-linked genetic syndrome, wherein pathogenic variants of the gene dystrophin cause a lack or absence of a normally functioning dystrophin protein in muscle cells. DMD is a lethal disorder with the main cause of death either cardiac failure or respiratory demise, and, at present, there is no effective therapy to treat cardiac dysfunction in this disorder. Fullenkamp et al. (2024) developed an *in vitro* modelling system using patient-derived induced pluripotent stem cell (iPSC) cardiomyocytes that are grown on a dynamic membrane, in an effort to simulate cardiac muscle contraction in the context of a pathogenic variant of the dystrophin gene. This unique model system incorporates patient-specific variants and provides a robust platform for drug and small-molecule therapy screening. The authors assessed several biomarkers in their model system to demonstrate that addition of annexin A6 – a scaffolding protein important in several critical cellular homeostatic mechanisms – improved the DMD cardiac myocyte phenotype.

Similar to DMD are dystroglycanopathies, a set of muscular dystrophies, some of which involve brain and eye abnormalities. Dystroglycanopathies are associated with variants in *DAG1* or enzymes involved in its glycosylation. The *DAG1* gene encodes two subunits, highly glycosylated α-dystroglycan (DG) and the transmembrane β-DG, which are primarily responsible for linking the cytoskeleton to the extracellular matrix. The study by Tan et al. (2024) produced and characterised a mouse model harbouring a C667F variant in *DAG1*, which was initially observed in a family with two affected individuals with muscle–eye–brain disease. Only one-third of the homozygous mice developed to birth and these mice developed a late-onset myopathy characterised by histology and muscle function. Interestingly, their brain and eye structures were normal but they had altered protein composition in the blood–brain and blood–retina barrier. Due to its incomplete penetrance, this mouse model has utility in studying disease mechanisms associated with variants in *DAG1* during embryogenesis and postnatal development. This study also demonstrates how patient-specific variants can be functionally characterised.

Another exciting illustration of how gene identification can be linked to functional analysis is provided by Tophkhane et al. (2024) in their study of Robinow syndrome, a rare disorder characterised by short stature, genital anomalies and craniofacial dysmorphism. Robinow syndrome is caused by variants in Wnt signalling pathway genes, including the transmembrane receptor *FZD2*. Here, Tophkhane et al. (2024) analyse the functions of two missense variants of *FZD2*, which are variants of uncertain significance. They use chicken embryo models in which they overexpress wild-type or variant forms of *FZD2* and are able to show that these disease variants fail to activate a SOX9 promoter reporter, whereas they cause ectopic Wnt activation, which perturbs craniofacial tissue morphogenesis.

Some rare diseases can have more complex aetiology that incorporates both genetic and environmental factors. TANGO2 deficiency disorder (TDD) is a rare neurodegenerative disorder that is caused by loss-of-function variants in TANGO2 and is accompanied by potentially lethal metabolic crises that are triggered by diet or illness. In a Perspective article, Michael Sacher, Chiara Gamberi and colleagues discuss the complex aetiology of TDD and how vitamin B5 supplementation could be a powerful treatment (Sacher et al., 2024). In a study of the rare liver cancer cholangiocarcinoma, by Alexander et al. (2024), the relationship between several cancer-associated genes and liver injury was uncovered in mice. The authors found that when inactivation of *Smad4* is combined with liver injury, it causes the tissue to become cancerous. This informs the mechanism of early tumorigenesis, which could lead to earlier diagnosis and improved therapies.

Overall, these articles demonstrate that rare genetic conditions can now be more accurately and precisely modelled *in vitro* and *in vivo*. This is essential to thoroughly understand the mechanisms of these diseases and, therefore, to develop strategies to improve clinical outcomes.

## Accelerating the journey from genotype to phenotype

There are many challenges in studying rare disease, and one of the major roadblocks is interpreting the functional effects of variants in disease-associated genes. Biochemical investigations of gene variants remain the gold standard in understanding the function of the encoded protein and the pathogenetic mechanism of disease, but with the wealth of genetic data generated from population and patient cohorts, the functional analysis of variants needs to be accelerated. Thankfully, technological advances are expanding capabilities in rare disease research. The ‘At a Glance’ poster by Kaiyue Ma, Monkol Lek and colleagues proposes an array of high-throughput *in vitro* assays for functional analysis of gene variants (Ma et al., 2024), and *in silico* techniques are also becoming increasingly informative and accurate. For example, Matsell et al. (2024) employed a combination of *in vitro* and *in silico* tools to investigate variants of the *ATP8A2* gene, which is associated with the ultra-rare cerebellar ataxia, impaired intellectual development and disequilibrium syndrome 4 (CAMRQ4).

Another significant development for interpreting gene–disease associations is the standardised phenotyping of mouse models performed by the International Mouse Phenotyping Consortium (IMPC) coupled with Mammalian Phenotype Ontology (MP). The study by Cacheiro et al. (2024) applied the PhenoDigm algorithm comparing recent IMPC phenotype data to human disease using Human Phenotype Ontology. They were able to find matches for approximately half of the mouse mutants to their corresponding human ortholog-associated disease. This represents a twofold increase from when this analysis was last performed in 2019, highlighting the increase and improvement of the MP associated with IMPC mouse models. The IMPC resource has been utilized in over 125 publications, providing insights into disease mechanisms of 109 genes, showcasing its utility and impact in Mendelian gene discovery.

In contrast to the mouse, the nematode worm *Caenorhabditis elegans* is a simple yet elegant genetic model for human disease that can easily be used to screen many thousands of individuals for genetic mutations. Bai et al. (2024) engineered a precise genetic model for a lipodystrophy that occurs in humans, which caused the worm embryos to die because their eggshells lacked the proper lipid structure. Although implementing a diet with certain fat supplementation rescued the ability of the eggs to hatch, the mechanism for this rescue was not clear. Thus, the authors carried out an unbiased forward genetic suppressor screen to identify mutations that rescued the effect of the lethal mutation. This not only provides a better understanding of the mechanism of lipodystrophies, but also an avenue to develop rational treatment strategies.

It is evident that there are many approaches to accelerate and improve our interpretation of disease-associated gene variants. By continuing to advance these techniques and employ complementary strategies, we can expedite translational discoveries in rare disease research.

## Rare disease research enhances our understanding of fundamental biological processes

Studying rare disease can also inform us of fundamental biological processes, such as development. In a Review article, Kate Rauen and William Tidyman discuss how investigating the mechanisms of RASopathies – a group of rare disorders associated with dysregulation of the RAS pathway – can enhance our understanding of skeletal muscle development (Rauen and Tidyman, 2024). RASopathies can also cause neurodevelopmental symptoms, so Bjorklund et al. (2024) expressed a hyperactive MEK1 mutant in cortical excitatory neurons of mice to determine how its activity regulates forebrain development and impacts RASopathy-associated phenotypes. The authors found that these mice presented with deficits in skilled motor learning as well as reduced axon outgrowth. This demonstrated that hyperactivation of MEK1 in cortical excitatory neurons plays a role in RASopathy pathogenesis and, more generally, in the essential process of long-range axonal outgrowth.

This concept of learning fundamental biology from rare disease research is also pertinent for congenital heart defects. Berger, Gerstner and colleagues used the African claw-toed frog *Xenopus laevis* to test the hypothesis that truncating variants in FBRSL1 cause congenital heart anomalies (Berger et al., 2024). They used CRISPR/Cas9 genome editing to demonstrate that FBRSL1 is required during development of the first heart field, which could be rescued by a human isoform of FBRSL1, but not with the disease variant. McKay et al. (2024) also tracked mice with a knockout of *Alms1* through clinically relevant developmental stages to better understand the role of this gene in cardiac development and cardiac complications in people with Alström syndrome. Furthermore, Hampl et al. (2024) used mouse genetics and pharmacological perturbations in chicken embryos to identify requirements for a putative transcriptional kinase, CDK13, in facial outgrowth and palatogenesis in CHDFIDD (congenital heart defects, facial dysmorphism and intellectual disorders) syndromes. Their study also proposes a new role for CDK13 in the cytoplasm, beyond its suggested roles in transcription and RNA processing. Similarly, while investigating the role of ATP1A3 in the rare neurodevelopmental disorder alternating hemiplegia of childhood, Fujii et al. (2024) uncovered that this protein interacts with key heat stress proteins to regulate protein synthesis and ensure mitochondrial stability under heat stress conditions.

Moving up the biological scale from individual proteins, Salazar Leon et al. (2024) investigated the role of a specific cell type – the Purkinje cells in the cerebellum of the brain – in the movement disorder cerebellar ataxia. They found that these cells play a critical role in sleep regulation in a mouse model, which is important as severe sleep disruptions can also be a symptom of cerebellar ataxia. Therefore, rare disease research is not only necessary to improve clinical outcomes for affected individuals, but it can also enhance our understanding of development and other essential biological processes.

## Inclusive and equitable rare disease research

Due to the inherent nature of rare diseases, patients and their families face unique challenges. Low numbers of individuals with a disease often means that diagnosis and treatment are more difficult, and funding for research to develop new diagnostic and therapeutic approaches is also more limited. In our interview with Veronica Kinsler (Kinsler, 2024), she outlined the difficulties in conducting rare disease research from the perspective of a clinician scientist. Importantly, she highlights the immense benefits of working closely with patients and patient advocacy groups to drive her research in a patient-focused manner. In this vein, in our ‘The Patient's Voice’ interviews, we spoke with Ian Stedman, who co-founded the Canadian Autoinflammatory Network (https://www.autoinflammatory.ca/). Ian focuses his advocacy work on bringing people with different autoinflammatory diseases together to encourage broad cross-disciplinary approaches that will improve healthcare and research (Stedman, 2024). We also spoke with Harsha Rajasimha, who co-founded the Organization for Rare Diseases India (ORDI; https://ordindia.in/) (Rajasimha et al., 2014) and founded the Indo US Organization for Rare Diseases (IndoUSrare; https://www.indousrare.org/). Here, he discussed the importance of cross-border collaborations and how to ensure equity in rare disease research (Krishnaraj and Rajasimha, 2024).

Prioritising equity in scientific and clinical advances is especially pertinent for the rare disease community. In a Perspective article, Tom Fox and Claire Booth explore how we can ensure that advanced gene therapies are accessible for patients with rare diseases across the globe (Fox and Booth, 2024). In another Perspective article, Dana Marafi outlines how investigating founder mutations in the Arab world and beyond can improve health in isolated communities and augment our overall understanding of rare disease (Marafi, 2024). As individuals with a rare disease and their families can often feel under-represented, it is essential that we make efforts to connect with these communities across the world and not isolate patients further based on geography, race or ethnicity.

## Conclusions

The Research and Resources & Methods articles in this Special Issue employ a variety of model systems to investigate a diverse range of rare diseases. By investigating these individual disorders using differing and complementary approaches, common themes can appear in the broad and heterogenous field of rare disease research. International and cross-disciplinary collaboration and patient partnership are essential for the progression of rare disease research. Importantly, these articles are openly accessible and are accompanied by our ‘Research Simplified’ summaries for all interested researchers, clinicians, patients and their families. Refined model systems and innovative technology are already enabling tangible clinical progress for many patients. We look forward to future research advances that will undoubtedly enrich DMM's ongoing subject collection and the field as a whole.

## References

[DMM052009C1] Akinyele, O., Munir, A., Johnson, M. A., Perez, M. S., Gao, Y., Foley, J. R., Nwafor, A., Wu, Y., Murray-Stewart, T., Casero, R. A., Jr et al. (2024). Impaired polyamine metabolism causes behavioral and neuroanatomical defects in a mouse model of Snyder–Robinson syndrome. *Dis. Model. Mech.* 17, dmm050639. 10.1242/dmm.050639 38463005 PMC11103582

[DMM052009C2] Alexander, W. B., Wang, W., Hill, M. A., O'Dell, M. R., Ruffolo, L. I., Guo, B., Jackson, K. M., Ullman, N., Friedland, S. C., McCall, M. N. et al. (2024). *Smad4* restricts injury-provoked biliary proliferation and carcinogenesis. *Dis. Model. Mech.* 17, dmm050358. 10.1242/dmm.050358 38415925 PMC10924230

[DMM052009C3] Bai, X., Smith, H. E. and Golden, A. (2024). Identification of genetic suppressors for a BSCL2 lipodystrophy pathogenic variant in *Caenorhabditis elegans*. *Dis. Model. Mech.* 17, dmm050524. 10.1242/dmm.050524 38454882 PMC11051982

[DMM052009C4] Berger, H., Gerstner, S., Horstmann, M.-F., Pauli, S. and Borchers, A. (2024). Fbrsl1 is required for heart development in *Xenopus laevis* and *de novo* variants in *FBRSL1* can cause human heart defects. *Dis. Model. Mech.* 17, dmm050507. 10.1242/dmm.050507 38501224 PMC11128277

[DMM052009C5] Bjorklund, G. R., Rees, K. P., Balasubramanian, K., Hewitt, L. T., Nishimura, K. and Newbern, J. M. (2004). Hyperactivation of MEK1 in cortical glutamatergic neurons results in projection axon deficits and aberrant motor learning. *Dis. Model. Mech.* 17, dmm050570. 10.1242/dmm.050570 PMC11247507 38826084

[DMM052009C6] Cacheiro, P., Pava, D., Parkinson, H., VanZanten, M., Wilson, R., Gunes, O., International Mouse Phenotyping Consortium and Smedley, D. (2024). Computational identification of disease models through cross-species phenotype comparison. *Dis. Model. Mech.* 17, dmm050604. 10.1242/dmm.050604 38881316 PMC11247498

[DMM052009C7] Fox, T. A. and Booth, C. (2024). Improving access to gene therapy for rare diseases. *Dis. Model. Mech.* 17, dmm050623. 10.1242/dmm.050623 38639083 PMC11051979

[DMM052009C8] Fujii, F., Kanemasa, H., Okuzono, S., Setoyama, D., Taira, R., Yonemoto, K., Motomura, Y., Kato, H., Masuda, K., Kato, T. A. et al. (2024). ATP1A3 regulates protein synthesis for mitochondrial stability under heat stress. *Dis. Model. Mech.* 17, dmm050574. 10.1242/dmm.050574 38804677 PMC11247502

[DMM052009C9] Fullenkamp, D. E., Willis, A. B., Curtin, J. L., Amaral, A. P., Dittloff, K. T., Harris, S. I., Chychula, I. A., Holgren, C. W., Burridge, P. W., Russell, B. et al. (2024). Physiological stress improves stem cell modeling of dystrophic cardiomyopathy. *Dis. Model. Mech.* 17, dmm050487. 10.1242/dmm.050487 38050701 PMC10820750

[DMM052009C10] Hampl, M., Jandová, N., Lusková, D., Nováková, M., Szotkowská, T., Čada, Š., Procházka, J., Kohoutek, J. and Buchtová, M. (2024). Early embryogenesis in CHDFIDD mouse model reveals facial clefts and altered cranial neurogenesis. *Dis. Model. Mech.* 17, dmm050261. 10.1242/dmm.050261 38511331 PMC11212636

[DMM052009C11] Hooper, K. M. and Hmeljak, J. (2023). Supporting the translation of multiscale research in rare disease. *Dis. Model. Mech.* 16, dmm050495. 10.1242/dmm.050495 37737037 PMC10538288

[DMM052009C12] Kinsler, V. A. (2024). Piecing together the mosaic of rare skin diseases: an interview with Veronica Kinsler. *Dis. Model. Mech.* 17, dmm050636. 10.1242/dmm.050636 38235593 PMC10820732

[DMM052009C13] Krishnaraj, P. and Rajasimha, H. K. (2024). Cross-border rare disease advocacy: Preethi Krishnaraj interviews Harsha Rajasimha. *Dis. Model. Mech.* 17, dmm050672. 10.1242/dmm.050672 38270284 PMC10846506

[DMM052009C14] Ma, K., Gauthier, L. O., Cheung, F., Huang, S. and Lek, M. (2024). High-throughput assays to assess variant effects on disease. *Dis. Model. Mech.* 17, dmm050573. 10.1242/dmm.050573 38940340 PMC11225591

[DMM052009C15] Marafi, D. (2024). Founder mutations and rare disease in the Arab world. *Dis. Model. Mech.* 17, dmm050715. 10.1242/dmm.050715 38922202 PMC11225585

[DMM052009C16] Marshall, G. F., Fasol, M., Davies, F. C. J., Le Seelleur, M., Fernandez Alvarez, A., Bennett-Ness, C., Gonzalez-Sulser, A. and Abbott, C. M. (2024). Face-valid phenotypes in a mouse model of the most common mutation in *EEF1A2*-related neurodevelopmental disorder. *Dis. Model. Mech.* 17, dmm050501. 10.1242/dmm.050501 38179821 PMC10855229

[DMM052009C17] Matsell, E., Andersen, J. P. and Molday, R. S. (2024). Functional and *in silico* analysis of ATP8A2 and other P4-ATPase variants associated with human genetic diseases. *Dis. Model. Mech.* 17, dmm050546. 10.1242/dmm.050546 38436085 PMC11073571

[DMM052009C18] McKay, E. J., Luijten, I., Broadway-Stringer, S., Thomson, A., Weng, X., Gehmlich, K., Gray, G. A. and Semple, R. K. (2024). Female Alms1-deficient mice develop echocardiographic features of adult but not infantile Alström syndrome cardiomyopathy. *Dis. Model. Mech.* 17, dmm050561. 10.1242/dmm.050561 38756069 PMC11225586

[DMM052009C19] Medeiros, D., Ayala-Baylon, K., Egido-Betancourt, H., Miller, E., Chapleau, C., Robinson, H., Phillips, M. L., Yang, T., Longo, F. M., Li, W. et al. (2024). A small-molecule TrkB ligand improves dendritic spine phenotypes and atypical behaviors in female Rett syndrome mice. *Dis. Model. Mech.* 17, dmm050612. 10.1242/dmm.050612 38785269 PMC11139040

[DMM052009C20] Nguengang Wakap, S., Lambert, D. M., Olry, A., Rodwell, C., Gueydan, C., Lanneau, V., Murphy, D., Le Cam, Y. and Rath, A. (2020). Estimating cumulative point prevalence of rare diseases: analysis of the Orphanet database. *Eur. J. Hum. Genet.* 28, 165-173. 10.1038/s41431-019-0508-0 31527858 PMC6974615

[DMM052009C21] Rajasimha, H. K., Shirol, P. B., Ramamoorthy, P., Hegde, M., Barde, S., Chandru, V., Ravinandan, M. E., Ramchandran, R., Haldar, K., Lin, J. C. et al. (2014). Organization for rare diseases India (ORDI) – addressing the challenges and opportunities for the Indian rare diseases’ community. *Genet. Res.* 96, e009. 10.1017/S0016672314000111 PMC7044965 25579084

[DMM052009C22] Rauen, K. A. and Tidyman, W. E. (2024). RASopathies – what they reveal about RAS/MAPK signaling in skeletal muscle development. *Dis. Model. Mech.* 17, dmm050609. 10.1242/dmm.050609 38847227 PMC11179721

[DMM052009C23] Sacher, M., DeLoriea, J., Mehranfar, M., Casey, C., Naaz, A., Mackenzie, S. J. and Gamberi, C. (2024). TANGO2 deficiency disorder is predominantly caused by a lipid imbalance. *Dis. Model. Mech.* 17, dmm050662. 10.1242/dmm.050662 38836374 PMC11179719

[DMM052009C24] Salazar Leon, L. E., Brown, A. M., Kaku, H. and Sillitoe, R. V. (2024). Purkinje cell dysfunction causes disrupted sleep in ataxic mice. *Dis. Model. Mech.* 17, dmm050379. 10.1242/dmm.050379 38563553 PMC11190574

[DMM052009C25] Stedman, I. (2024). Igniting an autoinflammatory disease community: an interview with Ian Stedman. *Dis. Model. Mech.* 17, dmm050642. 10.1242/dmm.050642 38231149 PMC10820735

[DMM052009C26] Tan, R. L., Sciandra, F., Hübner, W., Bozzi, M., Reimann, J., Schoch, S., Brancaccio, A. and Blaess, S. (2024). The missense mutation C667F in murine β-dystroglycan causes embryonic lethality, myopathy and blood-brain barrier destabilization. *Dis. Model. Mech.* 17, dmm050594. 10.1242/dmm.050594 38616731 PMC11212641

[DMM052009C27] Tophkhane, S. S., Fu, K., Verheyen, E. M. and Richman, J. M. (2024). Craniofacial studies in chicken embryos confirm the pathogenicity of human *FZD2* variants associated with Robinow syndrome. *Dis. Model. Mech.* 17, dmm050584. 10.1242/dmm.050584 38967226 PMC11247504

